# Efficacy of behavioral interventions to improve maternal mental health and breastfeeding outcomes: a systematic review

**DOI:** 10.1186/s13006-022-00501-9

**Published:** 2022-09-05

**Authors:** Lacey Pezley, Kate Cares, Jennifer Duffecy, Mary Dawn Koenig, Pauline Maki, Angela Odoms-Young, Margaret H. Clark Withington, Manoela Lima Oliveira, Bernardo Loiacono, Jilian Prough, Lisa Tussing-Humphreys, Joanna Buscemi

**Affiliations:** 1 Department of Kinesiology and Nutrition, College of Applied Health Sciences, University of Illinois at Chicago, 1919 W. Taylor St, Chicago, IL 60612 USA; 2 Department of Psychiatry, University of Illinois at Chicago, Chicago, IL USA; 3 Department of Human Development Nursing Science, University of Illinois at Chicago, Chicago, IL USA; 4 College of Human Ecology, Cornell University, New York, NY USA; 5 Department of Psychology, DePaul University, Chicago, IL USA

**Keywords:** Behavioral interventions, Depression, Anxiety, Mental health, Breastfeeding, Chestfeeding, Lactation

## Abstract

**Background:**

Despite extensive benefits and high intentions, few mothers breastfeed exclusively for the recommended duration. Maternal mental health is an important underlying factor associated with barriers and reduced rates of breastfeeding intent, initiation, and continuation. Given evidence of a bidirectional association between maternal mental health and breastfeeding, it is important to consider both factors when examining the efficacy of interventions to improve these outcomes. The purpose of this manuscript is to review the literature on the efficacy of behavioral interventions focused on both maternal mental health and breastfeeding outcomes, examining the intersection of the two.

**Methods:**

This systematic review was completed using the Preferred Reporting Items for Systematic Reviews and Meta-Analyses (PRISMA) reporting guidelines. Studies were selected if they were available in English, used primary experimental design, and used a behavioral intervention type to examine maternal mental health and breastfeeding outcomes. Articles were identified from PubMed, CINAHL, Embase, and PsycINFO from database inception to 3 March 2022. Study quality was assessed using the Cochrane Risk of Bias tool. Results were synthesized by intervention success for 1. Mental health and breastfeeding, 2. Breastfeeding only, 3. Mental health only, and 4. No intervention effect. PROSPERO CRD42021224228.

**Results:**

Thirty interventions reported in 33 articles were identified, representing 15 countries. Twelve studies reported statistically significant positive effect of the intervention on both maternal mental health and breastfeeding; most showing a decrease in self-report depressive and/or anxiety symptoms in parallel to an increase in breastfeeding duration and/or exclusivity. Common characteristics of successful interventions were a) occurring across pregnancy and postpartum, b) delivered by hospital staff or multidisciplinary teams, c) offered individually, and d) designed to focus on breastfeeding and maternal mental health or on breastfeeding only. Our results are not representative of all countries, persons, experiences, circumstances, or physiological characteristics.

**Conclusions:**

Interventions that extend the perinatal period and offer individualized support from both professionals and peers who collaborate through a continuum of settings (e.g., health system, home, and community) are most successful in improving both mental health and breastfeeding outcomes. The benefits of improving these outcomes warrant continued development and implementation of such interventions.

**Systematic review registration:**

PROSPERO CRD42021224228.

**Supplementary Information:**

The online version contains supplementary material available at 10.1186/s13006-022-00501-9.

## Background

Despite the many benefits of breastfeeding, few mothers breastfeed for the recommended duration. All major health and professional organizations, including the World Health Organization, American Academy of Pediatrics, and the United States (U.S.) Departments of Agriculture and Health and Human Services (Dietary Guidelines for Americans) [1–3] recommend exclusive breastfeeding for the first 6 months of a child’s life. Recommendations for continued breastfeeding, in combination with appropriate complementary foods, range from at least 1 to 2 years, as long as desired by both the mother and child [1, 2]. However, epidemiological data show that few mothers breastfeed to 1 year. According to the Centers for Disease Control and Prevention’s 2020 Breastfeeding Report Card, while 84% initiated breastfeeding, 58% were breastfeeding at 6 months postpartum, and only 35% were breastfeeding at 12 months [4]. Importantly, these low breastfeeding rates at 1 year persist despite high rates of intention to breastfeed. In the U.S., 80% of mothers intend to breastfeed in some capacity, and of those, more than 85% intend to exclusively breastfeed for at least 3 months; however, only one third (32%) of mothers achieve their intended breastfeeding goals [5].

Discrepancies between breastfeeding recommendations and actual breastfeeding duration have been explored. Reported barriers include: neonatal intensive care unit admission of the newborn, pain or discomfort when breastfeeding, difficulty with latching, concerns with adequate milk supply, lack of professional lactation support, employment circumstances, unaccommodating childcare environments, and unsupportive social and cultural norms [6–9]. These barriers are further complicated by mental health disorders, which are common during pregnancy and the first 12 months after childbirth [10–12]. Specifically, research suggests the prevalence of perinatal anxiety disorders is at least 17%, approximately 7–20% of mothers experience clinical depression at some time during the perinatal period [13, 14], and up to 1 in 3 (34%) mothers report experiencing childbirth trauma, often leading to postpartum depression [15] and post-traumatic stress disorder [16]. Given the high prevalence of mental health disorders within the perinatal period, maternal mental health has been considered an important underlying factor associated with barriers and reduced rates of breastfeeding intention, initiation, exclusivity, and continuation [10–12].

Research has consistently shown that maternal mental health disorders are associated with poorer breastfeeding outcomes. For example, prenatal anxiety is associated with reduced breastfeeding intention and postpartum anxiety is associated with reduced initiation, exclusivity, and duration of breastfeeding [17, 18]. In addition, childbirth trauma negatively affects initiation and continuation of breastfeeding [19, 20], and a strong association exists between perinatal depression and reduced breastfeeding intention, exclusivity, and duration [21]. Research has also shown that not engaging in breastfeeding or having a negative breastfeeding experience may increase the risk of postpartum depressive symptoms [22–24], while engaging in breastfeeding may protect against or ameliorate these symptoms [25, 26]. Given these associations, it has generally been accepted that the relationship between maternal mental health and breastfeeding is bidirectional, whereby mental health disorders may impede breastfeeding success and difficulty with or absence of breastfeeding may predict postpartum depression and anxiety [17, 21, 22, 24, 26]. Shared risk factors (e.g., self-efficacy, lack of social support, disrupted sleep) and overlapping neuroendocrine mechanisms (e.g., regulation of oxytocin, prolactin, serotonin, and cortisol) of mental health disorders and breastfeeding are thought to explain this bidirectional relationship [27, 28]. Therefore, it is important to consider both factors when examining the efficacy of interventions to improve these outcomes.

Indeed, many interventions have been developed and implemented to improve mental health or breastfeeding outcomes. However, to our knowledge, there are no published systematic reviews that examine the efficacy of behavioral interventions that focus on *both* maternal mental health and breastfeeding outcomes. Therefore, the purpose of this manuscript was to systematically review the literature on the efficacy of behavioral interventions which included outcomes of both maternal mental health (depression, anxiety, and childbirth trauma) *and* breastfeeding (intention, initiation, duration, exclusivity, knowledge, and self-efficacy). By examining behavioral interventions that assessed both outcomes, we may better understand the intersection of the two and determine intervention components that affect them. Since mental health and breastfeeding have not historically been studied together, gaining a better understanding of how they overlap may lend insight to a more wholistic approach to care, improving our understanding of how to create and reform best practices which can improve the short and long-term health of the mother, child, and family unit.

## Methods

This systematic review was completed using the Preferred Reporting Items for Systematic Reviews and Meta-Analyses (PRISMA) reporting guidelines. Details of the protocol for this systematic review were registered on PROSPERO and can be accessed at https://www.crd.york.ac.uk/prospero/display_record.php?RecordID=224228 [29]. We used the Covidence [30] software to manage title and abstract screening, full-text screening, quality assessment, and data extraction processes. The team consisted of five reviewers (B.L., J.P., K.C., L.P., and M.C.W.). Throughout these processes, each publication was independently evaluated by two reviewers using the conventional double-screening method. When discrepancies arose, all reviewers met and came to a consensus.

### Data sources and search methodology (identification)

Using an a priori research protocol, relevant articles were identified from PubMed, CINAHL, Embase, and PsycINFO from database inception to 3 March 2022, in consultation with a senior research librarian at the University of Illinois at Chicago. The general search terms used included variants of breastfeeding, depression, anxiety, and trauma. The full search strategy can be found in an additional file (see Additional file 1). The search terms were organized by database and included both database-specific Subject Heading and Keyword searches. A total of 6195 studies were identified using this search strategy.

### Study selection (screening and eligibility)

**Fig. 1:**
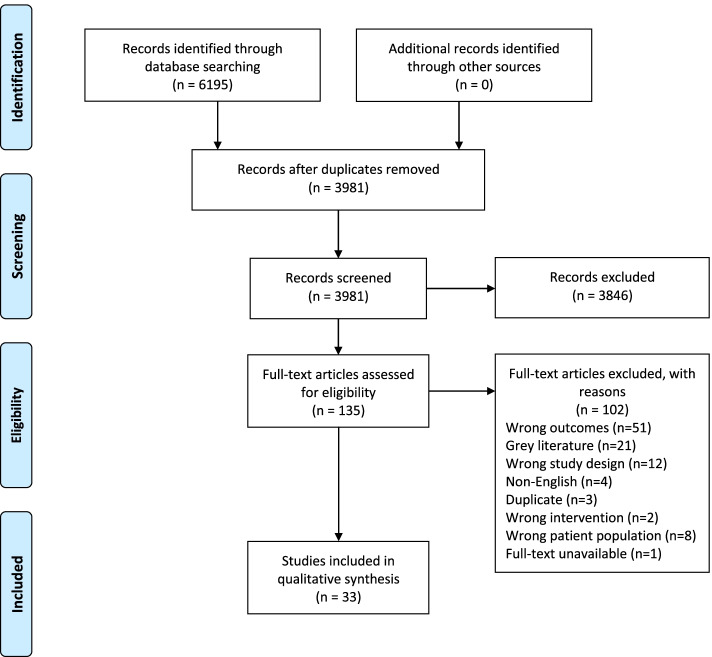
PRISMA Flow Diagram of Search Strategy and Study Selection

After automatic deduplication was completed in Covidence, a total of 3981 studies were available to be screened at the title and abstract level. For the purpose of this systematic review, empirical studies that assessed the effectiveness of behavioral interventions for improving maternal mental health and breastfeeding outcomes were included; the intervention itself did not have to focus on both factors, but inclusion of both outcomes was required. In this review, behavioral interventions, rather than medical, were included to home in on behavioral components that can be applied in future intervention efforts. Maternal mental health outcomes were depression, anxiety, and childbirth trauma. Various aspects of breastfeeding were considered, including intention, initiation, duration, exclusivity (feeding only human milk, not any other foods or liquids, except for medications or vitamin and mineral supplements), milk onset and volume, perceived milk supply, knowledge, and self-efficacy. Only articles available in English, those with primary experimental research design, and studies which used a behavioral intervention type were considered. Studies were included regardless of sample size or measurement type. A total of 135 full-text studies were assessed for eligibility, of which, 33 studies were included. A PRISMA flow diagram of the search strategy and study selection was generated (Fig. 1).

### Quality assessment and data extraction

The quality of each study was independently assessed in Covidence using the Cochrane Risk of Bias [31] template. Risk was assessed for each publication by two independent reviewers for each of the following domains: sequence generation, allocation concealment, blinding of participants and personnel, blinding of outcome assessors, incomplete outcome data, and selective outcome reporting. Using an a priori data extraction protocol and the Covidence software, independent reviewers extracted pertinent data including authors and country, research design, participant characteristics (age, race/ethnicity, income, parity, mode of delivery, past breastfeeding experience, and mental health history), intervention description, breastfeeding outcomes (intention, initiation, duration, exclusivity, milk onset and volume, knowledge, self-efficacy), and mental health outcomes (depression, anxiety, childbirth trauma) when available. All results that were compatible with each outcome domain were sought. Although measure of effect varied for each study, we synthesized our main outcome (breastfeeding and mental health) results based on reported statistical significance (*p* < 0.05). No additional analyses (e.g., subgroup, sensitivity, certainty assessment) were performed.

### Data synthesis

Relevant data from the final publications were extracted and organized in table format (Table 1). To examine the main outcomes, publications were synthesized by intervention success: 1. Successful interventions for mental health and breastfeeding outcomes, 2. Successful interventions for breastfeeding outcomes only, 3. Successful interventions for mental health outcomes only, and 4. Interventions with no effect. Within each of these sections of Table 1, publications were organized by the timing of the intervention (e.g., pregnancy, during the hospital stay at or around the time of birth, postpartum, and across both pregnancy and postpartum). When sample characteristics were not reported (NR), this was indicated.

**Table 1:** Summary of published behavioral interventions to improve breastfeeding and maternal mental health outcomes (*n* = 30 interventions)

*Reference (year), Country*	*Sample Characteristics*	*Intervention Components*	*Intervention Timing*	*Method of Intervention Delivery / Design Focus*	*Breastfeeding Results*	*Mental Health Results*
**Successful Interventions for Mental Health and Breastfeeding Outcomes (*n*** **= 12)**						
Zhao (2020, 2021, 2021), China [32–34]	*N* = 91 (I); 91 (C) Age (mean): 30.8 (I); 30.2 (C) Race/ethnicity: 100% Chinese Income (household, monthly): $898–1196: 19% (I); 26% (C) $1197- ≥ 1495: 81% (I); 74% (C) Parity: 100% (I and C) primiparous Mode of birth: 66% (I); 67% (C) vaginal MH status/history: 98.8% (I); 97.6% (C) with no depression history	I: individualized mixed management intervention consisting of 4 in-person psycho-educational sessions (1 hr.) focused on perinatal MH and BF C: standard obstetric care	Pregnancy	Psychiatrist and IBCLC Individual BF, MH	**Initiation:** ↑ at 3 days PP* **Exclusivity**: ↑ at 3 and 42 days and 3 mos. PP*; No difference at 6 mos. PP **Self-Efficacy:** ↑ at 3- and 42-days PP* (BSES) **Effective BF Behavior:** ↑ at 3- and 42-days PP*	**Depressive Symptoms:** ↓ at 3- and 42-days PP* (EPDS); ↓ at 3 and 6 mos. PP* (MINI)
Bigelow (2014), Canada [35]	*N* = 26 (I); 51 (C) Age (mean): 32.1 (I); 28.8 (C) Race/ethnicity: 100% (I); 98% (C) non-Hispanic White Income: NR Parity (mean): 1.1 (I); 1.2 (C) Mode of birth: NR MH status/history: NR	I: skin-to-skin contact 6 hrs. Per day during infant’s first week of life, then 2 hrs. Per day through 1 mos. PP (> 4000 min. total) C: < 4000 min. Total skin-to-skin in the 1st mos. PP	Hospital Stay	Hospital Staff Individual BF	**Duration:** ↑ at 1, 2, and 3 mos. PP* **Exclusivity:** ↑ at 1, 2, and 3 mos. PP*	**Depressive Symptoms:** ↓ at 1 wk. PP*; no difference between groups at 1, 2, and 3 mos. PP (EPDS)
Liu (2018), China [36]	*N* = 130 (I); 130 (C) Age: 40.8% (I); 41.9% (C) between 18 and 34 Race/ethnicity: NR Income (household, annual): ≤4000: 20% (I and C) > 4000: 30% (I and C) Parity: 34.2% (I); 36.5% (C) primiparous Mode of birth: 100% (I and C) cesarean MH status/history: NR	I: health education intervention developed according to the Health Belief Model that encouraged milk expression within 1 hr. after cesarean birth; expressed milk via hospital grade electric double pump every 2–3 hrs. For 20–30 min. C: standard education by obstetric nurses	Hospital Stay	Hospital Staff Individual BF	**Milk Onset:** Earlier lactation time* **Milk Volume:** ↑ during 24, 24 to 48, and 48 to 72 hours* **Exclusivity:** ↑ at 42 days PP* **Breast Swelling:** ↓ at 3 days PP*	**Anxiety Symptoms:** ↓ at 3 days PP* (STAI)
Song (2017), China [37]	*N* = 60 (I); 60 (C) Age (mean): 30.8 (I); 31.3 (C) Race/ethnicity: NR Income: NR Parity: 100% (I and C) primiparous Mode of birth: 100% (I and C) cesarean MH status/history: NR	I: psychological nursing care consisting of appropriate and timely support was offered before, during, and after cesarean birth C: standard nursing care	Hospital Stay	Hospital Staff Individual BF	**Initiation:** ↑ at 1 day before discharge* **Lactation yield:** ↑ at 3 days PP*	**Depressive Symptoms:** ↓ at 1 day before discharge* (Scale of Depression Score)
Çiftçi and Arikan (2011), Turkey [38]	*N* = 32 (I); 30 (C) Age: NR Race/ethnicity: NR Income: NR Parity: NR Mode of birth: NR MH status/history: NR	I: one-on-one BF training (1 hr.) + 5 in-home visits from 2 wks. Before returning to work through 6 mos. PP C: 5 in-home visits, but no BF training	Postpartum	Lactation Specialist Individual BF, MH	**Duration:** ↑ at 6 mos. PP* **Duration per feeding:** ↑ at 6 mos. PP* **Exclusivity:** ↑ at 3, 4, 5, and 6 mos. PP* **Frequency per day/night:** ↑*	**Anxiety Symptoms:** ↓ at 6 mos. PP* (STAI)
Franco-Antonio (2022), Spain [39]	*N* = 44 (I); 44 (C) Age (mean): 32.4 (I); 33.3 (C) Race/ethnicity: NR Income (household, annual): <$14,000: 16% (I); 11% (C) >$24,000: 18% (I); 16% (C) Parity: 34% (I and C) primiparous Mode of birth: Vaginal: 91% (I); 93% (C) Vaginal with forceps/vacuum: 9% (I); 7% (C) MH status/history: NR	I: Single brief motivational interviewing session immediate PP to promote BF and telephone booster session at 1 mos. PP C: Education leaflet for successful BF	Postpartum	Midwives Individual BF	**Duration:** ↑ at 3 mos. PP* **Self-Efficacy:** No difference between groups at 3 mos. PP (BSES-SF)	**Depressive Symptoms:** ↓ at 3 mos. PP* (EPDS> 10)
Vidas (2011), Croatia [40]	*N* = 50 (I); 50 (C) Age: NR Race/ethnicity: NR Income: NR Parity: NR Mode of birth: NR MH status/history: NR	I: autogenic training as a relaxation technique taught for 12 wks. in small groups from 2 to 6 mos. PP in BFing persons C: no autogenic training	Postpartum	Research Staff Group BF	**Duration:** ↑ at 6 mos. PP* **Exclusivity:** ↑ at 6 mos. PP*	**Depressive Symptoms:** ↓ at 6 mos. PP* (EPDS) **Anxiety Symptoms:** ↓ at 6 mos. PP* (STAI)
Buultjens (2018), Australia [41]	*N* = 10 (I); 8 (C) Age (mean): 32.6 (I); 31.9 (C) Race/ethnicity: 90% (I); 62.5% (C) White Income (household, annual): <$35,654–71,308: 30% (I); 62.5% (C) >$71,309: 70% (I); 37.5% (C) Parity: 100% primiparous Mode of birth: NR MH status/history: 70% (I); 75% (C) with no history of MH difficulties	I: psycho-educational group program met weekly for 2 hrs. From 3rd trimester through 8 wks. PP C: standard care with addition of a weekly phone call	Across Pregnancy and Postpartum	Multidiscipli-nary Team Group BF, MH	**Exclusivity:** No difference at 2–5 wks. PP; ↑ 12–14 wks. PP*	**Depressive Symptoms:** No difference between groups at 34–36 wks. Gestation; ↓ at 38–40 wks. Gestation, 2–5 wks., 5–8 wks., and 12–14 wks. PP* (EPDS)
Gureje (2019), Nigeria [42]	*N* = 452 (I); 234 (C) Age (mean): 24.5 (I); 24.9 (C) Race/ethnicity: NR Income: NR Parity: 56% (I); 49% (C) primiparous Mode of birth: NR MH status/history: EPDS score ≥ 12, but no psychotic symptoms, bipolar disorder, or suicidality	I: stepped-care treatment using a manualized psychological intervention package; 8 psychological sessions during pregnancy; 4–8 weekly interventions sessions starting at 6 wks. PP; pharmacotherapy as needed C: basic specifications of MH Gap Action Program; no structured sessions; no stepped-care procedure	Across Pregnancy and Postpartum	OB Care Providers Individual MH	**Exclusivity:** ↑ at 6 mos. PP*	**Depressive Symptoms:** No difference between groups at 6 mos. PP; ↓ 12 mos. PP* (EPDS)
Johnston (2004, 2006), United States [43, 44]	*N* = 439 (2004); 239 (I); 104 (C) Age (mean): NR (2004); 32.5 (I); 30.9 (C) Race/ethnicity: NR (2004); 78.6% (I); 80.6% (C) White Income (household, annual): NR (2004) < 40,000: 17.1% (I); 13.6% (C) 40,000–75,000: 45.7% (I); 38.8% (C) > 75,000: 37.2% (I); 47.6% (C) Parity: 53.4% primiparous (2004); 52.9% (I and C) primiparous Mode of birth: NR MH status/history: NR	I: risk-based intervention (Healthy Steps) focused on developmental and behavioral services starting at 1 wk. PP; includes home visits and phone support I: Healthy Steps plus 3 additional antenatal home visits and phone support starting between 16 and 20 wks. Gestation (PrePare) C: standard care	Across Pregnancy and Postpartum	Healthcare Provider Individual BF, MH	**Initiation:** No difference between groups (2004); ↑ for all intervention groups* (2006) **Duration:** ↑ at 3 and 6 mos. PP for all intervention groups*	**Depressive Symptoms:** ↓ at 3 mos. PP, but ↑ at 30 mos. PP for all intervention groups* (modified CES-D)
Kenyon (2016), United Kingdom [45]	*N* = 662 (I); 662 (C) Age (mean): 21.8 (I); 21.5 (C) Ethnicity: 48% (I and C) British Income: NR Parity: 100% nulliparous Mode of birth: NR MH status/history: 15% with clinical diagnosis of past or present mental illness (I and C)	I: pregnancy outreach worker service providing individual case management with home visits offered from < 28 wks. Gestation through 6 wks. PP. Prenatal services supported healthy lifestyle choices and social/emotional/mental difficulties. PP services supported BF and infant care. C: standard UK care	Across Pregnancy and Postpartum	Peer Support Individual MH	**Duration:** No difference between groups at 6–8 wks. PP **Self-efficacy:** No difference between groups at 8–12 wks. PP (Pearlin Mastery Scale) **Bonding:** ↑ at 8–12 wks. PP*	**Depressive Symptoms:** No difference between groups; ↓ at 8–12 wks. PP for those with more severe baseline symptoms* (EPDS)
Lutenbacher (2018), United States [46]	N = 91 (I); 87 (C) Age (mean): 30.4 (I); 28.7 (C) Race/ethnicity: 100% Hispanic Income (household, annual): <$15,000: 96.7% (I); 96.5% (C) Parity: median of 2 children in home Mode of birth: NR MH status/history: NR	I: Maternal Infant Health Outreach Worker model consisting of monthly home visits (1 hr.) and periodic group gatherings focusing on maternal concerns, healthy lifestyle, child development and attachment, and BF offered from < 26 wks. Gestation through 6 mos. PP C: distribution of printed educational material about maternal and infant health and development	Across Pregnancy and Postpartum	Peer Support Individual and Group BF, MH	**Initiation:** No difference between groups **Duration:** No difference between groups through 6 mos. PP **Exclusivity:** ↑ through 6 wks. PP* **Self-efficacy:** ↑ at 2 wks., 2 and 6 mos. PP* (BSES-SF)	**Depressive Symptoms:** ↓ at 2 wks., 2 and 6 mos. PP* (EPDS) **Stress:** ↓ at 2 wks., 2 and 6 mos. PP* (PSI)
**Successful Interventions for Breastfeeding Outcomes Only (*n*** **= 6)**						
Akbarzadeh (2017), Iran [47]	*N* = 50 (I); 50 (C) Age (mean): 23.9 (I); 24.4 (C) Race/ethnicity: NR Income: NR Parity: 100% primiparous Mode of birth: NR MH status/history: NR	I: Behavior-Change Model involving 4 weekly group BF educational sessions (90 min.) based on BASNEF in late pregnancy C: standard care	Pregnancy	Hospital Staff Group BF	**Knowledge:** ↑ immediately, 1 and 3 mos. After the intervention*	**Depressive Symptoms:** No difference between groups at 1 and 3 mos. After intervention (Zung Self-Rating Depression Scale)
Langer (1998), Mexico [48]	*N* = 361 (I); 361 (C) Age (mean): 22.5 (I and C) Race/ethnicity: NR Income: NR Parity: 93.1% (I); 90.6% (C) primiparous; no previous vaginal birth Mode of birth: Vaginal with forceps: 2.8% (I); 3.4% (C) Cesarean: 23.8% (I); 27.2% (C) MH status/history: NR	I: doula support involving continuous emotional, informational, and physical support through active labor; BF support during the immediate PP C: standard care	Hospital Stay	Doula Individual BF, MH	**Exclusivity:** ↑ at 1 mos. PP* **Knowledge:** ↑ behaviors that promote BF at 1 mos. PP*	**Anxiety Symptoms:** No difference between groups at immediate PP (STAI)
Saatsaz (2016), Iran [49]	*N* = 52 (foot); 52 (hand+foot); 52 (C) Age (mean): 27 (foot); 26.7 (hand+foot); 27.8 (C) Race/ethnicity: NR Income: NR Parity: 100% primiparous Mode of birth: 100% cesarean MH status/history: NR	I: foot massage (5 min./limb) given 4 hrs. After the last dose of analgesic following cesarean birth I: hand and foot massage (5 min./limb) given 4 hrs. After the last dose of analgesic following cesarean birth C: no massage	Hospital Stay	Massage Therapist Individual MH	**Frequency:** ↑ after cesarean birth in all intervention groups compared to control*	**Anxiety Symptoms:** No difference between groups after cesarean birth (STAI)
Ahmed (2016), United States [50]	*N* = 49 (I); 57 (C) Age (mean): 29.9 (I); 29.2 (C) Race/ethnicity: White: 73.5% (I); 67.9% (C) Black: 18.4% (I); 28.3% (C) Hispanic: 2% (I); 5.3% (C) Income (individual, annual): < 10,000: 8.2% (I); 19.3% (C) ≥50,000: 71.4% (I); 47.4% (C) Parity: 42.9% (I); 57.9% (C) primiparous Mode of birth: 73.5% (I); 73.7% (C) vaginal MH status/history: NR	I: online interactive BF monitoring system with automatic feedback via notifications for any reported BF issues within the first mos. PP C: standard care	Postpartum	Lactation Specialist Individual BF	**Exclusivity:** No difference between groups at discharge; ↑ at 1, 2, and 3 mos. PP* **Daily Frequency (Intensity):** ↑ at 1, 2, and 3 mos. PP*	**Depressive Symptoms:** No difference between groups at 1, 2, and 3 mos. (EPDS)
Sainz Bueno (2005), Spain [51]	*N* = 213 (I); 217 (C) Age: 54.9% (I); 53.1% (C) between 20 and 30 Race/ethnicity: NR Income: NR Parity: 18.2% (I); 19% (C) primiparous Mode of birth: 100% (I and C) vaginal MH status/history: NR	I: early hospital discharge (24 hrs.), monitored at home by a nurse for 24–48 hrs., in-clinic follow up at 7–10 days PP, and telephone consultation at 1, 3, and 6 mos. PP C: standard hospital discharge (48h hrs.), in-clinic follow up at 7–10 days PP, and telephone consultation at 1, 3, and 6 mos. PP	Postpartum	Home Health Nurse Individual BF, MH	**Duration:** ↑ at 3 mos. PP*; no difference at 1 wk., 1 mos., 6 mos., or > 9 mos. PP	**Depressive Symptoms:** No difference between groups at 1wk. and 1 mos. PP (HAD)
Hans (2018), United States [52]	*N* = 156 (I); 156 (C) Age (mean): 18.5 (I); 18.3 (C) Race/ethnicity: African American: 43.6% (I); 46.2% (C) Latina/Hispanic: 39.1% (I); 35.9% (C) Income: 100% low income (I and C) Parity: 97.4% (I); 98.7% (C) primiparous Mode of birth: 23.2% (I); 21.5% (C) cesarean MH status/history: CES-D score of 14.2 (I); 13.8 (C) at baseline	I: doula-home-visiting program with weekly prenatal home visits by a home visitor and/or community doula; doula support during labor and birth, and through 6 wks. PP C: case management	Across Pregnancy and Postpartum	Community Doulas and Peers Individual BF, MH	**Initiation:** ↑ at the hospital stay* **Duration:** No difference between groups at 3 mos. PP	**Depressive Symptoms:** No difference between groups at 3 wks. and 3 mos. PP (CES-D)
**Successful Interventions for Mental Health Outcomes Only (*n*** **= 5)**						
Rossouw (2021), South Africa [53]	N = 50 (I); 50 (C) Age (mean): 27.2 (I); 27.9 (C) Race/ethnicity: 90% (I); 88% (C) Black African Income: 100% living in a low-resource, low employment environment Parity: 21% (I); 33% (C) primiparous Mode of birth: NR MH status/history: NR	I: Community Health Worker program (30–90 min. Monthly visits) and incentive package (baby items, maternity pads, condoms) C: standard prenatal care	Pregnancy	Community health worker Individual BF, MH	**Intention to BF exclusively:** No difference between groups at 1 wk. PP	**Depressive Symptoms:** ↓ at 1 wk. PP* (EPDS)
Zhao (2017), China [54]	*N* = 176 (I); 176 (C) Age (mean): 30.4 (I); 30.6 (C) Race/ethnicity: NR Income (household, monthly): <$598–1196: 16% (I); 15.4% (C) $1197- ≥ 1495: 84% (I); 84.6% (C) Parity: 100% (I and C) primiparous Mode of birth: 54.3% (I); 43.1% (C) vaginal MH status/history: 98.9% (I); 99.4% (C) with no depression history	I: prenatal couple-separated psycho-educational group sessions (6 at 90 min. each) focused on maternal MH and family support; sessions 1–5 were for high-risk pregnant persons, while session 6 was for their partner C: standard obstetrical care	Pregnancy	Research Staff Group MH	**Exclusivity:** No difference between groups at 42 days PP	**Depressive Symptoms:** ↓ at 42 days PP* (EPDS)
Mohd Shukri (2019), Malaysia [55]	*N* = 33 (I); 31 (C) Age: 51.5% (I); 67.7% (C) between 26 and 30 Race/ethnicity: 90.9% (I); 96.8% (C) Malay Income (household, monthly): $360–1202: 54.5% (I); 54.8% (C) $1202 → 2405: 45.5% (I); 45.3% (C) Parity: 100% (I and C) primiparous Mode of birth: 75% (I and C) vaginal MH status/history: NR	I: relaxation therapy via audio-guided imagery protocol designed for BF persons provided in-home at 2, 6, and 12 wks. PP; instructed to listen during the subsequent 2 wks. C: no relaxation therapy	Postpartum	Research Staff Individual BF, MH	**Human milk Intake:** No difference between groups	**Anxiety Symptoms:** ↓ at 2 wks. PP*; no difference between groups at 6–8 wks. and 12–14 wks. PP (BAI) **Stress:** No difference between groups at 2 wks. PP; ↓ at 6–8 wks. and 12–14 wks. PP* (PSS) **Stress:** ↓ at 2 wks. PP* (hindmilk cortisol); no difference between groups at 6–8 wks. PP (milk cortisol)
Morrell (2000), United Kingdom [56]	*N* = 311 (I); 312 (C) Age (mean): 27.5 (I); 28 (C) Race/ethnicity: NR Income: 30% (I); 29% (C) receiving housing benefit Parity (mean): 1.9 (I); 1.8 (C) Mode of birth: Spontaneous vaginal: 68% (I); 73% (C) Elective cesarean: 8% (I); 7.7% (C) Emergency cesarean: 9.6% (I); 10.2% (C) Twin birth: 2.9% (I); 0.32% (C) MH status/history: NR	I: PP care at home by community midwives plus up to 10 in-home visits from a support worker for up to 3 hrs./day in the first 28 days PP C: PP care at home by community midwives	Postpartum	Community Midwife and PP Support Worker Individual BF, MH	**Exclusivity:** No difference between groups at 6 wks. and 6 mos. PP	**Depressive Symptoms:** ↓ at 6 wks. PP*; no difference between groups at 6 mos. PP (EPDS)
Montazeri (2020), Iran [57]	*N* = 35 (I); 35 (C) Age (mean): 28 (I and C) Race/ethnicity: NR Income: 17.1% (I); 14.3% (C) with insufficient household income Parity: NR Mode of birth: 23.5% (I); 13.3% (C) cesarean MH status/history: NR	I: In-person group journal therapy sessions (3 at 45–60 min. each) from the 3rd trimester to the end of pregnancy; telephone counseling sessions (3 at 15 min. each) from the 3rd trimester to 1 mos. PP C: routine care	Across Pregnancy and Postpartum	Research Staff Group and Individual MH	**Exclusivity:** No difference between groups at 2 and 4 mos. PP (Exclusive BF Checklist)	**Anxiety Symptoms:** ↓ at 6 wks. Post intervention, 2 and 4 mos. PP* (BAI)
**Interventions with No Effect (*n*** **= 7)**						
Rotheram-Fuller (2017), United States [58]	*N* = 99 (I); 104 (C) Age (mean): 28.5 (I); 27.8 (C) Race/ethnicity (language at home): 80% (I); 87% (C) Spanish Income (household, monthly): <$1000: 43.5% (I); 47.1% (C) $1001–2000: 42.4% (I); 41.3% (C) Parity: NR Mode of birth: NR MH status/history: 13.1% (I); 11.5% (C) with EPDS > 13 at baseline	I: home visiting or telephone support addressing maternal daily habits, BF, and depression; offered as needed during pregnancy C: standard care	Pregnancy	Peer Support Individual BF, MH	**Duration:** No difference between groups at 1 wk. through 6 mos. PP (IFI)	**Depressive Symptoms:** No difference between groups at 6 mos. (EPDS)
Tubay (2019), United States [59]	*N* = 61 (I); 68 (C) Age (mean): 28.1 (I); 27.8 (C) Race/ethnicity: White: 61% (I); 59% (C) Hispanic: 23% (I); 12% (C) Asian: 13% (I); 18% (C) African American: 10% (I); 15% (C) Income: E1-E5 Enlisted military rank: 35.7% (I); 31.8% (C) Parity: NR Mode of birth: 15% (I); 18% (C) unplanned cesarean MH status/history: NR	I: group prenatal care (CenteringPregnancy) starting ~ 16 wks. Gestation; 10 sessions (2 hrs.) across pregnancy C: standard prenatal care	Pregnancy	OB provider Group BF, MH	**Duration:** No difference between groups at 6 wks. PP	**Depressive Symptoms:** No difference between groups at 6 wks. PP (CES-D) **Anxiety Symptoms:** No difference between groups at 6 wks. PP (STAI)
Boulvain (2004), Switzerland [60]	*N* = 228 (I); 231 (C) Age (mean): 29 (I and C) Race/ethnicity: 31% (I); 30% (C) Swiss origin Income (household, annual): <$55,000: 27% (I); 24% (C) ≥$55,000: 57% (I and C) Parity: 60% (I); 57% (C) nulliparous Mode of birth: Spontaneous vaginal: 72% (I); 65% (C) Instrumental vaginal: 18% (I); 24% (C) Cesarean: 11% (I); 12% (C) MH status/history: no history of PP depression	I: home-based PP care by a midwife after shortened hospital stay (24–48 hrs.) C: hospital-based PP standard care lasting 4–5 days PP	Postpartum	Midwives Individual BF	**Duration:** No difference between groups at 7 days, 28 days, and 6 mos. PP	**Depressive Symptoms:** No difference at 7- and 28-days PP (EPDS)
Escobar (2001), United States [61]	*N* = 508 (I); 506 (C) Age (mean): 29 (I); 29.1 (C) Race/ethnicity: White: 48.8% (I); 50.6% (C) Hispanic: 21.7% (I); 21.2% (C) Asian/Pacific Islander: 23.2% (I); 22.4% (C) Income (household, annual): >$60,000: 50.2% (I); 53.3% (C) Parity 1: 46.6% (I); 45.4% (C) Mode of birth: 100% vaginal MH status/history: NR	I: home health nurse visits (60–90 min.) starting 48 hrs. After hospital discharge C: hospital-based follow-up anchored in group visits	Postpartum	Home Health Nurse Individual and Group BF, MH	**Duration:** No difference between groups at 2 wks. PP	**Depressive Symptoms:** No difference between groups at 2 wks. PP (CES-D)
Lieu (2000), United States [62]	*N* = 580 (I); 583 (C) Age (mean): 27.9 (I); 27.8% (C) Race/ethnicity: White: 62.9% (I); 58.8% (C) Hispanic: 13.4% (I); 11.5% (C) Income (household): 71.1% (I); 72.7% (C) above 200% of federal poverty level Parity: 39% (I); 39.3% (C) primiparous Mode of birth: NR MH status/history: NR	I: home visits (60–90 min.) starting within 48 hrs. After discharge C: standard individual PP clinic follow-up	Postpartum	Home Health Nurse Individual BF, MH	**Duration:** No difference between groups at 2 and 12 wks. PP	**Depressive Symptoms:** No difference between groups at 2 wks. PP (CES-D)
Nikodem (1993), South Africa [63]	*N* = 83 (I); 79 (C) Age (mean): 25.4 (I); 24.5 (C) Race/ethnicity: NR Income (monthly): <$68.25: 75.3% (I); 69.5% (C) Parity: 37.3% (I); 48.1% (C) primigravida Mode of birth: NR MH status/history: NR	I: audiovisual intervention featuring BF and health education videos within 72 hrs. After birth C: no audiovisual intervention	Postpartum	Hospital Staff Individual BF	**Duration**: No difference between groups at 6 wks. PP **Exclusivity:** No difference between groups at 6 wks. PP	**Depressive Symptoms:** No difference between groups at 6 wks. PP (Pitt’s Depression Questionnaire)
Galland (2017), New Zealand [64]	*N* = 802 Age (mean): 32 Ethnicity: 77.9% European Income (household, annual): >$46,645: 50.6% Parity: 48% primiparous Mode of birth: NR MH status/history: EPDS within normal range at baseline	I: sleep intervention with a single antenatal education group session (1 hr.) followed by a home visit at 3 wks. PP with an infant sleep training specialist I: Food, Activity and BF (FAB) intervention with BF education and support antenatally and at 1 wk. and 4 mos. PP provided by an IBCLC; physical activity support at 3 mos. PP I: combined sleep and FAB intervention C: standard care	Across Pregnancy and Postpartum	Sleep Specialist, IBCLC Individual and Group BF, MH	**Exclusivity:** No difference between groups at 4 and 6 mos. PP	**Depressive Symptoms:** No difference between groups at 4 mos. PP (EPDS)

*BAI* Beck Anxiety Inventory, *BASNEF* beliefs, attitudes, subjective norms and enabling factors, *BF* breastfeeding; *BSES-SF* Breastfeeding Self-Efficacy Scale-Short Form, *C* Control, *CES-D* Center for Epidemiological Study-Depressive Symptomatology Scale, *EPDS* Edinburgh Postpartum Depression Scale, *HAD* Hospital Anxiety and Depression Scale, *IFI* Infant Feeding Intention scale, *IBCLC* International Board Certified Lactation Consultant, *I* Intervention, *MH* mental health, *MINI* Mini-International Neuropsychiatric Interview; *NR* not reported, *OB* obstetrician, *PSI* Parenting Stress Index-Short Form, *PP* postpartum, *PSS* Perceived Stress Scale, *RCT* randomized controlled trial, *SPSQ* The Swedish Parenthood Stress Questionnaire, *s/p* status post, *STAI* Spielberger State Trait Anxiety Inventory

↑, increased; ↓, decreased

*, statistical significance (*p* < 0.05) with outcome direction according to the intervention group(s) relative to the control group

## Results

### General description

A total of 33 articles met the criteria for inclusion in this review. Two articles [43, 44] describe data from the same study and an additional three articles describe data from another study [32–34], for a total of 30 unique interventions. Table 1 provides a summary of sample characteristics, intervention components, and mental health and breastfeeding outcomes of the studies included. Overall, 29 of the studies were randomized controlled trials (RCT) and one study used alternate-allocation for ‘randomization’ [41]. Studies were published between 1993 and 2022, with a majority (20/30, 67%) being published in the past 10 years.

Of the 30 interventions included in this review, eight were conducted in the U.S. [43, 44, 46, 50, 52, 58, 59, 61, 62], four in China [32–34, 36, 37, 54], three in Iran [47, 49, 57], two each from South Africa [53, 63], Spain [39, 51], and the United Kingdom [45, 56], and one each from Canada [35], Switzerland [60], Australia [41], Turkey [38], New Zealand [64], Nigeria [42], Mexico [48], Malaysia [55], and Croatia [40]. Of the eight studies conducted in the U.S., five (63%) had a sample primarily consisting of white non-Hispanic participants [43, 44, 50, 59, 61, 62], two studies had primarily Hispanic and/or Spanish-speaking participants [46, 58], and one study had primarily Black and Hispanic participants [52].

Sample size varied greatly across studies from 18 to 1324 participants. Eight interventions were conducted in first-time parents only [32–34, 37, 41, 45, 47, 49, 54, 55]. Five studies did not state the parity of the sample [38, 40, 57–59]. Fourteen of the studies did not report mode of birth as a sample characteristic. Three reported 100% of participants had a vaginal birth [39, 51, 61] and three reported 100% of participants had a cesarean birth [36, 37, 49]. Income level varied greatly among study samples and was reported differently from study to study, household vs. individual and annual vs. monthly. A total of 10 studies did not report income as a sample characteristic.

To examine the efficacy of these behavioral interventions on mental health and breastfeeding outcomes, results are presented and synthesized into four categories: 1. Successful interventions for mental health and breastfeeding outcomes, 2. Successful interventions for breastfeeding outcomes only, 3. Successful interventions for mental health outcomes only, and 4. Interventions with no effect. The intervention timing, method of intervention delivery, and design focus are presented in Table 1.

### Successful interventions for mental health and breastfeeding outcomes

Twelve of the 30 studies reported statistically significant positive effect of the intervention on both maternal mental health and breastfeeding outcomes. Successful interventions included psychoeducational group programs [32–34, 41], relaxation therapy [40], skin-to-skin contact between mother and infant [35], psychological nursing [37], motivational interviewing [39], a health and infant care education program [36], stepped-care psychological treatment [42], peer support with home visits [45, 46], breastfeeding training with home visits [38], and risk-based treatment with home visits [43, 44].

Common characteristics among the successful interventions for mental health and breastfeeding outcomes were a) occurring across both pregnancy and the postpartum period (5/12, 42%) [41–46], b) delivered by hospital staff (3/12, 25%) [35–37] or by multidisciplinary teams of mental health and lactation specialists (3/12, 25%) [32–34, 41, 42], c) offered individually rather than in a group setting (9/12, 75%), and d) designed to focus on both breastfeeding and maternal mental health (5/12, 45%) [32–34, 38, 41, 43, 44, 46] or focused primarily on breastfeeding only (5/12, 45%) [35–37, 39, 40].

In regard to breastfeeding outcomes, eight studies reported breastfeeding exclusivity as an outcome and all eight indicated a statistically significant increase in exclusivity in the intervention group compared to the control, with assessment time points ranging from 3 days to 6 months postpartum [32–36, 38, 40–42, 46]. Breastfeeding duration was measured in eight studies, five of which indicated a statistically significant increase in duration at 3, 6, or 9 months postpartum in the intervention group compared to the control [35, 38–40, 43, 44]. Milk output/volume was measured in four studies and all four indicated a statistically significant increase in volume within the first 3 days to 2 weeks postpartum in the intervention group versus control [36, 37]. Initiation of breastfeeding was measured in four studies, three of which indicated a statistically significant increased rate of initiation among the intervention participants compared with control [32, 37, 44]. Breastfeeding self-efficacy was measured in four studies, two of which indicated a statistically significant enhanced self-efficacy between 3 days to 6 months postpartum in intervention versus control participants [32–34, 46]. Earlier milk onset [36], decreased breast swelling [36], greater levels of effective breastfeeding behavior (e.g., noticing changes in breast fullness, visualizing and hearing baby swallowing, etc.) [32–34] and increased mother-infant bonding [45] were reported among intervention versus control participants in these studies as well.

Regarding mental health outcomes, most studies reported depressive symptoms as an outcome (10/12, 83%). All (12/12, 100%) studies indicated a statistically significant decrease in the level of depressive symptoms at time points ranging from birth to 12 months postpartum among the intervention compared to the control participants. Although one study reported a decrease at 3 months postpartum, they showed more depressive symptoms at 30 months postpartum in the intervention group compared to control [44]. Symptoms of anxiety were measured in three studies and all reported lower levels across time points of 3 days to 6 months postpartum [36, 38, 40]. One study also reported a dose response of breastfeeding frequency, where the higher the frequency, the lower maternal anxiety levels became [38]. Stress was reported in one study and found lower levels of stress at 2 weeks and 2 and 6 months postpartum among the intervention participants [46].

### Successful interventions for breastfeeding outcomes only

Six of the 30 studies reported statistically significant positive effect of the intervention on breastfeeding, but not maternal mental health outcomes. Successful interventions included doula support [48, 52], early hospital discharge with home-based postpartum care [51], massage therapy [49], an online interactive breastfeeding monitoring system with real-time support from a lactation specialist [50], and breastfeeding education group sessions [47].

Common characteristics among the successful interventions for breastfeeding outcomes only were a) occurring during the hospital stay at or around the time of birth (2/6, 33%) [48, 49] or during the postpartum period only (2/6, 33%) [50, 51], b) delivered by doulas (2/6, 33%) [48, 52], c) offered in an individual setting (5/6, 83%) [48–52], and d) designed to focus on both breastfeeding and maternal mental health (3/6, 50%) [48, 51, 52].

Two studies reported breastfeeding exclusivity as an outcome and both indicated a statistically significant increase in exclusivity in the intervention group compared to the control, with time points ranging from 1 to 3 months postpartum [48, 50]. Breastfeeding duration was measured in two studies, with one indicating a statistically significant increase in duration at 3 months postpartum among the intervention participants, but not at 1 week, one, six, or greater than 9 months postpartum [51]. The other study found no difference between groups for breastfeeding duration at 3 months postpartum [52]. Breastfeeding frequency was reported in two studies. One showed increased daily frequency status post cesarean birth [49] and the other from 1 to 3 months postpartum [50] among intervention versus control participants. Two studies measured breastfeeding knowledge and found an increase at time points ranging from birth to three months postpartum [47, 48]. Greater rate of breastfeeding initiation among intervention compared to control participants was found in one study [52].

### Successful interventions for mental health outcomes only

Five of the 30 studies reported statistically significant positive effect of the intervention on maternal mental health, but not breastfeeding outcomes. Successful interventions included relaxation therapy [55], in-home postpartum support [56], prenatal psycho-educational group support [54], community health worker program plus incentive package [53], and journal therapy counseling [57].

Common characteristics among the successful interventions for mental health outcomes only were a) occurring during pregnancy only (2/5, 40%) [53, 54] or during the postpartum period only (2/5, 40%) [55, 56], b) delivered by research team members (3/5, 60%) [54, 55, 57], c) offered in an individual setting (3/5, 60%) [53, 55, 56], and d) designed to focus on both breastfeeding and maternal mental health (3/5, 60%) [53, 55, 56].

Three studies reported depressive symptoms as an outcome measured and each of these studies indicated a statistically significant decrease in the level of depressive symptoms at time points ranging from 1 week to 6 months postpartum among the intervention compared to the control participants [53, 54, 56]. Symptoms of anxiety were measured in two studies. One study reported a decrease at 2 weeks postpartum among intervention participants, but not at six and 12 weeks postpartum [55] and the other study reported a decrease at 2 and 4 months postpartum [57]. Stress was reported in one study using PSS and milk cortisol [55]. At 2 weeks postpartum, there were lower levels of stress as indicated by a decrease in hindmilk cortisol. Lower levels of stress were reported at six and 12 weeks postpartum according to the PSS among intervention participants [55].

### Interventions with no effect

Of the 30 studies included in this review, seven reported no statistically significant difference between intervention and control groups for mental health and breastfeeding outcomes. These interventions included home-based postpartum care [60–62], in-home antenatal support [58], group prenatal care [59], sleep intervention [64], and audiovisual postpartum breastfeeding education [63]. Common characteristics among the interventions with no effect were a) occurring during the postpartum only (4/7, 57%) [60–63], b) delivered by home healthcare providers (2/7, 29%) [61, 62] or by perinatal care providers (2/7, 29%) [59, 60], c) offered in an individual setting (4/7, 57%) [58, 60, 62, 63], and d) designed to focus on both maternal mental health and breastfeeding (5/7, 71%) [58, 59, 61, 62, 64].

### Risk of bias

The Cochrane Risk of Bias tool [31] was used to assess seven domains of bias (Table 2).

**Table 2:** Cochrane risk of bias for randomized controlled trials

Author (Year)	Random sequence generation	Allocation concealment	Blinding of participants and personnel	Blinding of outcome assessment	Incomplete outcome data addressed	Selective reporting	Other sources of bias^a^
Ahmed (2016) [50]							
Akbarzadeh (2017) [47]							
Bigelow (2014) [35]							
Boulvain (2004) [60]							
Buultjens (2018) [41]							
Çiftçi and Arikan (2011) [38]							
Escobar (2001) [61]							
Franco-Antonio (2022) [39]							
Galland (2017) [64]							
Gureje (2019) [42]							
Hans (2018) [52]							
Johnston (2004, 2006) [43, 44]							
Kenyon (2016) [45]							
Langer (1998) [48]							
Lieu (2000) [62]							
Liu (2018) [36]							
Lutenbacher (2018) [46]							
Mohd Shukri (2019) [55]							
Montazeri (2020) [57]							
Morrell (2000) [56]							
Nikodem (1993) [63]							
Rossouw (2021) [53]							
Rotheram-Fuller (2017) [58]							
Saatsaz (2016) [49]							
Sainz Bueno (2005) [51]							
Song (2017) [37]							
Tubay (2019) [59]							
Vidas (2011) [40]							
Zhao (2017) [54]							
Zhao (2020, 2021, 2021) [32–34]							

^a^Other sources of bias may include protocol adherence, other interventions avoided, sample size sufficiently large, eligible participants enrolled, funding and sponsorship bias

, yes;  , no;  , unclear

#### Random sequence generation (selection bias)

Adequate generation of a randomized sequence (low risk of selection bias) was described in 25 of the 30 RCTs. Two studies were at high risk for this bias [38, 59]. The method of randomization was not adequately described in three of the studies [40, 52, 58].

#### Allocation concealment (selection bias)

Adequate concealment of allocations prior to assignment (low risk of selection bias) was described in 19 of the 30 RCTs. Three studies were at high risk for this bias [35, 38, 53]. The method used to conceal the allocation sequence was not described in sufficient detail in eight of the studies.

#### Blinding of participants and personnel (performance bias)

Blinding was not always possible due to the nature of behavioral interventions. However, blinding of participants and personnel was ensured, or it was determined that the outcomes were not likely to be influenced by lack of blinding (low risk of performance bias) in 20 of the 30 RCTs. Four studies were at high risk for performance bias due to no or incomplete blinding [42, 53, 57, 58]. The method of blinding was not adequately described in six of the studies [40, 43–45, 47, 54, 63].

#### Blinding of outcome assessment (detection bias)

Blinding of the outcome assessment was ensured (low risk of detection bias) in 15 of the 30 RCTs. Two studies were at high risk for this bias [35, 36]. The method used to blind the outcome assessment was not described in sufficient detail in 13 of the studies.

#### Incomplete outcome data addressed (attrition bias)

The amount, nature, and handling of incomplete outcome data was appropriate (low risk of attrition bias) in 22 of the 30 RCTs. Four studies were at high risk for this bias [37, 41, 58, 63]. The method of blinding was not described in sufficient detail in four of the studies [36, 40, 47, 64].

#### Selective reporting (reporting bias)

Adequate description of the study’s pre-specified and expected outcomes (low risk of reporting bias) was provided in 22 of the 30 RCTs. Two studies were at high risk for this bias [47, 64]. This information was unclear or inadequate in six of the studies [38, 42, 49, 51, 52, 63].

#### Other sources of bias

Additional sources of potential bias assessed included protocol adherence, other interventions avoided, sample size sufficiently large, eligible participants enrolled, and funding and sponsorship bias. Low risk of other bias was found in 24 of the 30 RCTs. No studies were at high risk for this bias. This information was unclear or inadequate in six of the studies [37, 40, 43, 44, 49, 51, 58].

## Discussion

This review examined 33 articles which sought to test the effect of 30 unique interventions on both maternal mental health and breastfeeding outcomes. Over one-third (12/30, 40%) of the interventions were successful at improving both mental health and breastfeeding outcomes, six (20%) reported positive effects on breastfeeding only, five (17%) reported positive effects on mental health only, and almost a quarter (7/30, 23%) of interventions had no effect on mental health or breastfeeding outcomes. Interventions that improved both mental health and breastfeeding outcomes were more likely to span across pregnancy and the postpartum period, including at or around birth, while interventions demonstrating no effect or an effect on only mental health or breastfeeding mostly occurred in either pregnancy or the postpartum period alone. Successful interventions were also more likely to be delivered by a combination of hospital staff, mental health and lactation specialists, and peer support. These findings are consistent with evidence indicating that support provided concurrently throughout a continuum of settings (e.g., health system, home, and community) results in the largest positive impact of breastfeeding outcomes [65]. Research also suggests that communication and collaboration between providers from various disciplines can improve both maternal mental health and breastfeeding outcomes [66].

Across all outcome categories, most (22/30, 73%) interventions took place in an individual rather than group setting. In a qualitative review of breastfeeding experiences among those with postpartum depression, mothers indicated that non-judgmental, encouraging, timely, and individualized support from professionals that are competent in breastfeeding counseling is essential in their decision and ability to breastfeed [24].

Consistent with evidence of a bidirectional association between maternal mental health and breastfeeding, most successful interventions in this review showed an increase in breastfeeding duration and/or exclusivity in parallel to a decrease in self-report depressive and/or anxiety symptoms. Shared neuroendocrine mechanisms between mental health and breastfeeding are thought to play a role. In normal physiological conditions, the lactogenic hormones oxytocin and prolactin have mood-ameliorating effects; promoting feelings of relaxation during breastfeeding [67]. Breastfeeding is thought to lessen the stress response and enhance maternal mood. In fact, research has shown that salivary and plasma cortisol response to stress is suppressed in lactating individuals in situations of physical and psychological stress [68]. However, disruptions in the homeostasis of lactogenic hormones (i.e., low levels) can affect mood and breastfeeding success. For instance, physical or emotional stress is known to increase levels of salivary and plasma cortisol [17, 28], and higher cortisol levels can interfere with the regulation of oxytocin and prolactin [28] and have been associated with decreased milk volume [17]. Consistent with this mechanism, one study in this review reported increased milk volume with concurrent reduced levels of stress or anxiety [36]. It is important to note that while *perceived* concern of milk supply is one of the most common factors associated with early breastfeeding cessation and postpartum anxiety [20, 69, 70], none of the studies in this review assessed perceived milk supply.

Although intervention strategies varied greatly across studies, most interventions with a positive effect on mental health and breastfeeding were designed to focus on mental health and breastfeeding (5/12, 42%) or breastfeeding alone (5/12, 42%). This suggests that intervening on breastfeeding alone may be similarly effective as intervening on mental health and breastfeeding to improve both outcomes. Perhaps by supporting the breastfeeding experience, we are supporting something more; we are supporting the whole person and their community.

### Limitations

Several limitations of this review should be noted. First, after screening at the title and abstract phase, 3846 of the 3981 potentially eligible records were excluded indicating our search strategy may have been too broad. Next, the interventions took place across 15 different countries which makes it difficult to make direct comparisons given the varying policies and social environments that can affect maternal mental health and breastfeeding outcomes. Additionally, the majority (5/8, 63%) of U.S.-based samples in this review included white non-Hispanic participants only, making it difficult to consider the intersectional complexities of race, mental health, and breastfeeding. Future research must take an intersectional approach to understand how varying identities and compounding experiences of discrimination and oppression impact outcomes of mental health and breastfeeding. Previous breastfeeding experience, which could impact results, was only reported in four articles [39, 50, 61, 62]. In addition, parity was not consistent across studies and was not reported in many articles. Lactogenic hormone release is greater in multiparous mothers compared to primiparous, indicating that parity may be an important factor in the relationship between mental health and breastfeeding [25]. Many articles (21/30, 70%) did not report current or history of mental health difficulties within the study sample, which is a potential for unknown confounding. The varying follow-up time points and measurement strategies used across studies make it difficult to make direct comparisons as well. Next, childbirth experience continues to be underrepresented in the literature. Nearly half of the studies did not report mode of birth. Further, no studies were found examining childbirth-related trauma as an outcome. It is likely that the events that occur during labor and birth have an impact on breastfeeding outcomes, mostly due to the delay of lactogenesis II and the disruption of normal physiologic processes [71]. Childbirth trauma is also associated with increased risk of postpartum depression and post-traumatic stress disorder [15, 16]. Next. under- and over-nutrition may affect milk volume and composition. More specifically, there is data suggesting that obesity is associated with insufficient glandular development, reduced milk volume, dampened milk ejection reflex, suppressed lactation, and elevated depressive symptoms [71, 72], however, only four articles reported body mass index as a sample characteristic [39, 45, 49, 58]. Lastly, future research should examine how medications for mental health, in the presence and absence of behavioral intervention, may impact breastfeeding and mental health outcomes as well [73].

While not a limitation, it should be noted that only one intervention used digital-technology [50]. The Internet offers great potential in extending preventive services to individuals in the perinatal period since they address several key barriers to success such as limited access to professional support and lack of social support. Digital-technology interventions, which include the use of web-based content and interactions, text messaging, and social media, have been effective at reducing depressive symptoms and improving breastfeeding outcomes [24, 74]. Strengths of a digital approach to interventions for perinatal mothers include efficiency of time and resources, ability to reach geographically and racially diverse populations, and improved social support.

## Conclusions

This systematic review highlights the intersection of maternal mental health and breastfeeding. Both occur in complex settings that affect and can be affected by physiological, emotional, social, psychological, personal, cultural, and physical factors. Based on this review, interventions that extend across pregnancy and postpartum and offer individualized support from both professionals and peers who collaborate through a continuum of settings are most successful in improving both mental health and breastfeeding outcomes. The benefits of improving these outcomes warrant continued development and implementation of interventions that acknowledge and support the whole person and their community.

## Supplementary Information

**Additional file 1.** Search Strategy. Search terms used in PubMed, CINAHL, Embase, and PsycINFO.

## Data Availability

All data generated or analyzed during this study are included in this published article.

## Abbreviations

PRISMA: Preferred Reporting Items for Systematic Reviews and Meta-Analyses
RCT: randomized controlled trials
U.S.: United States
