# Supplementary material for: Efficacy of behavioral interventions to improve maternal mental health and breastfeeding outcomes: a systematic review
Source: Int Breastfeed J. 2022 Sep 5;17:67. doi: 10.1186/s13006-022-00501-9 (PMC9446548; doi:10.1186/s13006-022-00501-9)
Supplement: Supplementary file 1 — Additional file 1. Search Strategy. Search terms used in PubMed, CINAHL, Embase, and PsycINFO. [file 13006_2022_501_MOESM1_ESM.pdf]

Additional file 1. Search Strategy

Articles were identified from PubMed, CINAHL, Embase, and PsycINFO since database inception in consultation with a senior research librarian at the University of Illinois at Chicago. The search terms were be organized by database and included both database-specific Subject Heading and Keyword searches.

PubMed Search

((“Breast Feeding”[mesh] OR “Lactation”[mesh] OR “Breast Feeding”[tiab] OR “Breastfeeding”[tiab] OR “Lactation”[tiab] OR “Lactating”[tiab]) **AND** (“Mental Disorders”[mesh:NoExp] OR “Anxiety Disorders”[mesh] OR “Depressive Disorder”[mesh] OR “Depression”[mesh] OR “Trauma and stressor related disorders”[mesh] OR “Depression”[tiab] OR “Depressive”[tiab] OR “PPD”[tiab] OR “PND”[tiab] OR “Anxiety”[tiab] OR “Trauma”[tiab] OR “Traumatic”[tiab] OR “PTSD”[tiab]) **AND** (“Epidemiologic studies”[mesh] OR “Statistics and numerical data”[sh] OR “Controlled clinical trial”[pt] OR “randomized”[tiab] OR “1andomized”[tiab] OR “randomly”[tiab] OR “placebo”[tiab] OR “case control”[tiab] OR “cohort”[tiab] OR “cross sectional”[tiab] OR “follow up”[tiab] OR “observational”[tiab] OR “longitudinal”[tiab] OR “prospective”[tiab] OR “retrospective”[tiab] OR “investigated”[tiab] OR “analysis”[tiab] OR “statistics”[tiab] OR “data”[tiab])) **NOT** (“Animals”[Mesh] NOT (“Animals”[Mesh] AND “Humans”[Mesh]))

CINAHL Search

((MH “Breast Feeding+”) OR (MH “Lactation+”) OR AB(“Breast Feeding”) OR TI(“Breast Feeding”) OR AB(“Breastfeeding”) OR TI(“Breastfeeding”) OR AB(“Lactation”) OR

TI(“Lactation”) OR AB(“Lactating”) OR TI(“Lactating”)) **AND** ((MH “Behavioral and Mental Disorders”) OR (MH “Anxiety Disorders+”) OR (MH “Depression+”) OR (MH “Psychological Trauma+”) OR AB(“Depression”) OR TI(“Depression”) OR AB(“Depressive”) OR TI(“Depressive”) OR AB(“PPD”) OR TI(“PPD”) OR AB(“PND”) OR TI(“PND”) OR AB(“Anxiety”) OR TI(“Anxiety”) OR AB(“Trauma”) OR TI(“Trauma”) OR AB(“Traumatic”) OR TI(“Traumatic”) OR AB(“PTSD”) OR TI(“PTSD”)) **AND** ((MH “research by type and subject+”) OR AB(“randomized”) OR TI(“randomized”) OR AB(“randomized”) OR TI(“randomized”) OR AB(“randomly”) OR TI(“randomly”) OR AB(“placebo”) OR TI(“placebo”) OR AB(“case control”) OR TI(“case control”) OR AB(“cohort”) OR TI(“cohort”) OR AB(“cross sectional”) OR TI(“cross sectional”) OR AB(“follow up”) OR TI(“follow up”) OR AB(“observational”) OR TI(“observational”) OR AB(“longitudinal”) OR TI(“longitudinal”) OR AB(“prospective”) OR TI(“prospective”) OR AB(“retrospective”) OR TI(“retrospective”) OR AB(“investigated”) OR TI(“investigated”) OR AB(“analysis”) OR TI(“analysis”) OR AB(“statistics”) OR TI(“statistics”) OR AB(“data”) OR TI(“data”))

Embase Search

(‘Breast Feeding’/exp OR ‘Lactation’/exp OR ‘Breast Feeding’:ti,ab OR ‘Breastfeeding’:ti,ab OR ‘Lactation’:ti,ab OR ‘Lactating’:ti,ab) **AND** (‘Mental Disease’/de OR ‘Anxiety Disorder’/exp OR ‘Depression’/exp OR ‘Psychotrauma’/exp OR ‘Depression’:ti,ab OR ‘Depressive’:ti,ab OR ‘PPD’:ti,ab OR ‘PND’:ti,ab OR ‘Anxiety’:ti,ab OR ‘Trauma’:ti,ab OR ‘Traumatic’:ti,ab OR ‘PTSD’:ti,ab) **AND** (‘methodology’/exp OR ‘randomized’:ti,ab OR ‘randomised’:ti,ab OR ‘randomly’:ti,ab OR ‘placebo’:ti,ab OR ‘case control’:ti,ab OR ‘cohort’:ti,ab OR ‘cross sectional’:ti,ab OR ‘follow up’:ti,ab OR ‘observational’:ti,ab OR

‘longitudinal’:ti,ab OR ‘prospective’:ti,ab OR ‘retrospective’:ti,ab OR ‘investigated’:ti,ab OR ‘analysis’:ti,ab OR ‘statistics’:ti,ab OR ‘data’:ti,ab) **NOT** ([animals]/lim NOT [humans]/lim)

PsycINFO Search

(MAINSUBJECT.EXACT.EXPLODE(“breast feeding”) OR MAINSUBJECT.EXACT.EXPLODE(“Lactation”) OR AB,TI(“breast feeding”) OR AB,TI(“breastfeeding”) OR AB,TI(“Lactation”) OR AB,TI(“Lactating”)) **AND** (MAINSUBJECT.EXACT(“mental disorders”) OR MAINSUBJECT.EXACT.EXPLODE(“anxiety disorders”) OR MAINSUBJECT.EXACT.EXPLODE(“major depression”) OR MAINSUBJECT.EXACT.EXPLODE(“stress and trauma related disorders”) OR AB,TI(“depression”) OR AB,TI(“depressive”) OR AB,TI(“PPD”) OR AB,TI(“PND”) OR AB,TI(“anxiety”) OR AB,TI(“trauma”) OR AB,TI(“traumatic”) OR AB,TI(“PTSD”)) **AND** (MAINSUBJECT.EXACT.EXPLODE(“empirical methods”) OR AB,TI(“randomized”) OR AB,TI(“randomized”) OR AB,TI(“randomly”) OR AB,TI(“placebo”) OR AB,TI(“case control”) OR AB,TI(“cohort”) OR AB,TI(“cross sectional”) OR AB,TI(“follow up”) OR AB,TI(“observational”) OR AB,TI(“longitudinal”) OR AB,TI(“prospective”) OR AB,TI(“retrospective”) OR AB,TI(“investigated”) OR AB,TI(“analysis”) OR AB,TI(“statistics”) OR AB,TI(“data”))
